# Three-dimensional angular scattering simulations inform analysis of scattering from single cells

**DOI:** 10.1117/1.JBO.28.8.086501

**Published:** 2023-08-09

**Authors:** Kaitlin J. Dunn, Andrew J. Berger

**Affiliations:** University of Rochester, Institute of Optics, Rochester, New York, United States

**Keywords:** biomedical optics, cells, scattering, simulations, Mie theory, quantitative phase imaging

## Abstract

**Significance:**

Organelle sizes, which are indicative of cellular status, have implications for drug development and immunology research. At the single cell level, such information could be used to study the heterogeneity of cell response to drugs or pathogens.

**Aim:**

Angularly resolved elastic light scattering is known to be sensitive to changes in organelle size distribution. We developed a Mie theory-based simulation of angular scattering from single cells to quantify the effects of noise on scattering and size estimates.

**Approach:**

We simulated randomly sampled organelle sizes (drawn from a log normal distribution), interference between different organelles’ scattering, and detector noise. We quantified each noise source’s effect upon the estimated mean and standard deviation of organelle size distributions.

**Results:**

The results demonstrate that signal-to-noise ratio in the angular scattering increased with the number of scatterers, cell area, and exposure time and decreased with the size distribution width. The error in estimating the mean of the size distributions remained below 5% for nearly all experimental parameters tested, but the widest size distribution tested (standard deviation of 600 nm) reached 20%.

**Conclusions:**

The simulator revealed that sparse sampling of a broad size distribution can dominate the mismatch between actual and predicted size parameters. Alternative estimation strategies could reduce the discrepancy.

## 1 Introduction

Studying organelle sizes and dynamics in single cells provides unique insight compared with measurements that are averaged over ensembles of cells, for which conclusions can only be drawn about the entire cell population. For example, investigating individual cells allows scientists to compare the response of immune cells with specialized roles, to observe cell differentiation, and to study heterogeneity in tumors.^1^ The use of DNA sequencing and other single cell analysis techniques has spurred discoveries in drug development, cancer treatment,^1^ and immunology.^2^

Organelle behavior and morphology provide a window into assessing cellular state. In particular, mitochondrial dynamics are an indicator of metabolic demand and cell response; mitochondria undergo fission to a fragmented state during periods of rest when there is low respiratory activity and fuse into connected networks during times of high metabolic demand.^3^ In addition, the dysfunction of mitochondria has been linked to neurogenerative diseases,^4^ cancer,^5^ and metabolic conditions.^6^ This motivates the recent push for developing therapeutics that specifically target mitochondrial function^5^ and demonstrates that mitochondrial function can characterize a cell’s state. Other strong scatterers include lysosomes^7^ and lipid droplets. Lipid droplets play an important role in energy storage and cellular metabolism.^8^ Furthermore, lipid droplet sizes have been linked to distinct functional roles.^9^

Optical techniques such as microscopy have long been used to image cells, with fluorescence staining providing organelle-specific information. However, such labeling-based techniques have the potential to disrupt the very organelle function that is under study.^10^ Angularly resolved light scattering, a measurement of scattered intensity versus deflection angle, is a label-free technique that is sensitive to the size and refractive index of scatterers such as organelles. Mitochondria-sized organelle populations in cell ensembles have been sized using all or part of the forward-directed angular scattering range between 0 deg and 90 deg. For example, Wilson et al. used angular scattering to size populations of subcellular scatterers in ensembles of cells by assuming functional forms for the size distributions and fitting to their forward scattering using a Mie theory-based forward model.^11^^,^^12^ Boustany et al. quantified the increase in the ratio of low to high angle scattering as elongated organelles rounded up into spherical particles during calcium overload.^13^ In addition, Wilson et al. used a coated sphere model to explain changes in angular scattering as mitochondria swelled in response to oxidative stress.^12^ Angular light scattering is therefore a promising tool for studying mitochondrial dynamics as a marker of cell response and metabolic state.

Although non-nuclear organelle populations in cell ensembles have been sized using angular scattering, bringing this capability to the single-cell level requires a deeper understanding of the noise sources that influence angular scattering-based size estimates. Because knowledge of the ground truth of scatterer sizes in a single cell is difficult to obtain, we turn to simulations to gain insight into the ability to size organelle populations in single cells. The ground truth scatterer sizes are known in simulations, so the effects of various noise sources on the ability to accurately invert size distributions can be investigated.

### 1.1 Ideal Forward Model and Noise Sources

Before discussing what we define as noise sources, it is useful to describe what the ideal model of scattering from a single cell consists of. We define the idealized cell to contain spherical organelles suspended in a homogeneous cytosol, the diameters of which are represented by a size distribution. In the ideal forward scattering model, the only scattering comes from the index contrast between the organelles and the cytosol. Mie theory^14^ is used to model this scattering by summing the scattering from each organelle in intensity, assuming no interference. The sources of noise (i.e., deviation from this model) to be investigated in this simulation work are (1) “sampling noise,” or deviations from the assumed size distribution, (2) interference noise due to the coherent addition of scattering from spatially offset organelles, and (3) detector noise.

### 1.2 Simulating Measurements of Angular Scattering

To generate organelles for a cell simulation, a size distribution function is randomly sampled repeatedly to generate scatterer sizes. The scattered field is computed in the Fourier domain for each organelle, with a phase term added to account for its particular (x,y,z) location. The fields for all organelles are summed coherently and Fourier transformed to the spatial domain to arrive at the complex field scattered by the sample as would be measured in the object plane (z=0). Although sampling and interference noise only depend on the simulated sample, the effect of detector noise is dependent on the method of measuring angular scattering. Detector noise must be added to the intensity images in the domain at which measurements are made, and any digital processing steps must be simulated to determine how the noise propagates to the final angular scattering intensity measurement.

There are a variety of methods for measuring angular scattering, including direct intensity measurements such as goniometry or imaging the sample’s Fourier plane. A method known as Fourier transform light scattering (FTLS)^15^ is better suited for single cell analysis because it allows for digitally isolating a single cell or region of interest rather than measuring scattering from an entire field of view that could contain multiple cells. In FTLS, the complex field of the sample is measured with a quantitative phase imaging (QPI) technique, and the digitally selected region is Fourier transformed to the angular domain to obtain the angular scattering intensity. FTLS has been used for sizing beads with accuracy on the order of 10s of nanometers^16^ and measuring the angular scattering response of single HeLa cells,^17^ red blood cells,^18^ bacteria,^19^ and nuclei.^20^

Similarly, there are many QPI approaches that can be used to measure the complex field of light scattered by samples. To simulate detector noise in a way that matches our experimental system, we simulate the steps of a technique known as Fourier phase microscopy (FPM),^21^ which is described in further detail in Sec. 2. The effect of detector noise on angular scattering and size estimates can then be investigated by adding detector noise to the intensity measurements and processing them using the same procedure used for experimental data to compute angular scattering.

Simulations of angular light scattering from single cells were used to determine the effect of sampling noise, interference noise, and detector noise on the uncertainty in angular light scattering measurements. Mie theory-based fits to the angular scattering intensity were used to estimate the mean and standard deviation of the log-normal size distribution from which scatterer sizes were drawn. The effect of each noise source on the estimated size parameters was characterized by tuning the experimental parameters that vary the amount of each noise source and assessing the statistics of many simulations and fits. These simulations and fits lent insight into the degree to which each noise source limited the ability to accurately estimate scatterer sizes in single cells.

## 2 Methods

### 2.1 Simulation Overview

**Fig. 1:**
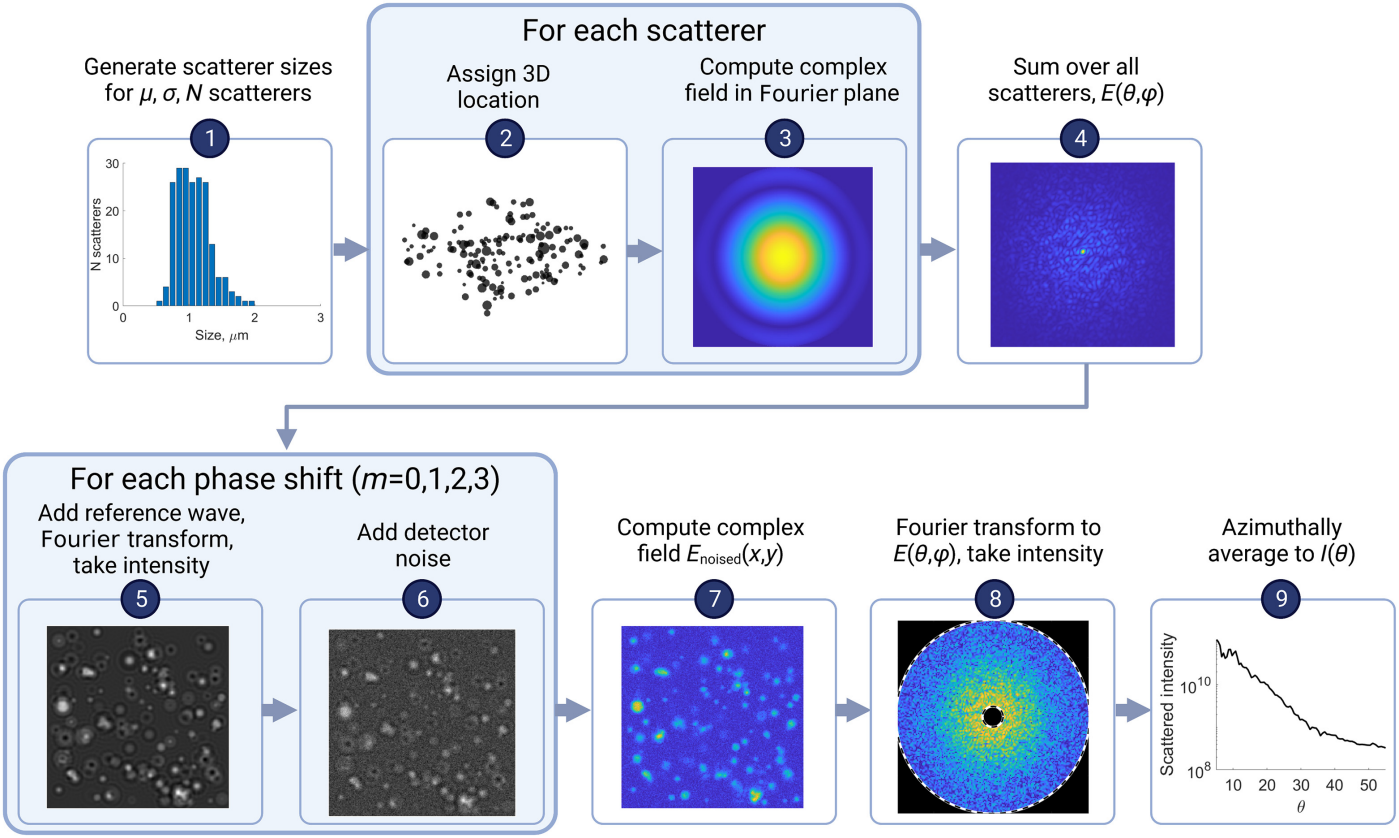
Block diagram of simulation procedure for generating a 1D angular scattering intensity I(θ) for an ensemble of N scatterers sampled from a log normal distributon n(μ,σ).

In the presented simulations of a single cell’s scattering, we model the organelles as an ensemble of homogeneous spheres suspended within a three-dimensional (3D) volume. The physics-based model then combines Mie theory with Fourier optics to compute the angular scattering. Figure 1 shows the simulation procedure. We use a single log-normal size distribution model, in which the user defines the number of scatterers N and the mean μ and width σ of the log normal. This is a simplification of real cells (e.g., ignoring larger scatterers such as the nucleus). The choice of a log-normal is motivated by Wilson et al.’s results,^11^ which showed that intact cellular scattering is well represented by two log-normal size distributions. In this work, we reverted to a single log normal to simplify quantification of the relationship between simulation parameters and size estimate accuracy. Scatterer sizes are obtained by randomly sampling the size distribution, and the locations are randomized within a user-defined region representative of a single cell’s volume. Mie theory^14^ is used to compute the complex field of the light scattered by each sphere upon illumination by a monochromatic, linearly polarized plane wave. For each scatterer, the phase of the field in the angular domain is modified to account for its 3D location by adding the appropriate tilt or defocus term. These complex fields are then summed coherently over all scatterers to compute the total scattered field, which accounts for the interference between organelles. This field is inverse Fourier transformed to the image domain, so the FPM imaging system can be simulated with detector noise. The noised data are then processed the same way as experimental data, by Fourier transforming back to the angular domain to compute the two-dimensional (2D) angular scattering. The individual steps are described in further detail in the remainder of this section.

The values used for simulations in this paper are given in Table 1 and were chosen to match experimental conditions in our angular scattering microscope described by Draham et al.^16^ and to realistically model scattering from single cells. The LED’s center wavelength λ, the photon flux density, and the exposure times were chosen to match our experimental setup. The minimum and maximum scattering angle are determined by the region phase shifted by the spatial light modulator (SLM) in our Fourier phase microscope and the numerical aperture of our microscope objective, respectively. The indices of refraction for the cytoplasm and organelles are from the literature.^22^ As was previously described, we used a single log normal functional form as a simple approximation of mitochondria-sized scatterers because mitochondria have a strong contribution to the scattering from intact cells.^12^ We simulated size distributions with a mean of 1.1  μm and widths between 100 and 600 nm, inspired by the fits to mitochondria-sized scatterers in Wilson et al.’s work.^11^^,^^12^ We used between 50 and 1000 scatterers^23^ to model mitochondria in a single cell and simulated cell widths of between 25 and 55  μm and axial thicknesses of 5  μm.

**Table 1:** Simulation parameters.

λ	780 nm
$t_{\exp}$	0.1 to 2 s
Photon flux density	30,000 photons/pixel/second
$\theta_{\min}$	5 deg
$\theta_{\max}$	55 deg
$n_{\text{cytoplasm}}$	1.37
$n_{\text{organelles}}$	1.4
n(d)	LN(μ,σ;d)
μ	1.1 μm
σ	100, 300, 600 nm
N scatterers	40 to 1000
Cell width	25 to 55 μm
Cell thickness	5 μm

### 2.2 Sampling Noise from Forward-Model Mismatch

A popular forward model choice for fitting size distributions with light scattering assumes a continuous distribution (e.g., a log normal). However, the finite number of scatterers contained within a cell limits the ability of the actual size distribution’s scattering to match that of the continuous model assumed for fitting. In addition, any random deviation from the assumed distribution’s functional form is a source of forward model mismatch between reality and the fitting model. The effects of having a finite number of scatterers and random deviations from the assumed scatterer model are hereafter referred to as “sampling noise,” which becomes especially important to explore at the single cell level due to the small number of organelles available to sample a size distribution. Therefore, the simulation tool allows for varying the number of scatterers, the functional form of the size distribution, and the ability to add randomness. This enables studying the effect of sampling noise on the accuracy with which size estimates can be inverted.

Any functional form of a size distribution n(d) can be used to generate scatterer sizes. To randomly sample the size distribution, we use the discrete inverse transform sampling method,^24^^,^^25^ which takes advantage of the fact that cumulative distribution functions range between 0 and 1. The goal is to obtain a discrete random variable D sampled N times from the probability distribution function p(d). The cumulative distribution function is given as

F(di)=∫0din^(x)dx, (1)

where $\hat{n}(x)$ is the normalized size distribution, i.e., the probability distribution function p(d). N uniformly distributed random numbers u are generated on the interval 0<u<1, and each random sample D is then defined as

$$D=d_{i},\;\text{if }F(d_{i-1})<u<F(d_{i}).$$ (2)

### 2.3 Interference Between Organelles

To simulate a cell’s scattering, an idealized model that assumes a collection of spherical organelles surrounded by a homogeneous cytoplasm is used. The complex field scattered by each organelle is modeled using Mie theory,^14^ which computes the complex field scattered from linearly polarized light incident on a homogeneous sphere located at the origin. We also include the appropriate tilt and defocus phase terms associated with the lateral (x,y) and axial (z) offset of each scatterer from the origin. The scattering from all organelles is summed coherently in field to yield the total angular scattering intensity, which includes the interference between the organelles. The angular-domain phase term for the j’th scatterer located at $\left(x_{j},y_{j},z_{j}\right)$ is

$$P_{j}=\exp(-i2\pi (f_{x}x_{j}+f_{y}y_{j}+\frac{z_{j}}{\lambda }\sqrt{1-{\left(\lambda f_{x}\right)}^{2}-{\left(\lambda f_{y}\right)}^{2}})),$$ (3)

where the $z_{j}$ term is the propagation transfer function that contains the defocus due to z-offset, the $f_{x}$ and $f_{y}$ terms are lateral spatial frequencies, the $x_{j},y_{j}$ terms are the phase tilt terms associated with a lateral shift in the image domain,^26^ λ is the wavelength in the medium, and $i=\sqrt{-1}$. Because the forward-scattered angular distribution of linearly polarized light scattered by organelle-sized spheres is highly azimuthally symmetric, it is useful to average the intensity within annular bins of constant θ. This performs some averaging over the interference noise and yields a one-dimensional (1D) vector of scattered intensity versus polar angle θ, or I(θ).

### 2.4 Fourier Phase Microscopy

Simulations of interference noise, computed by coherently combining the scattered field from each organelle as described in the previous section, are general to any angular scattering detection method. Simulating the effects of detector noise is more specific to the imaging modality. For techniques that require complex field measurement, different interferometry techniques and the computational processing required for obtaining the complex field will affect the propagation of noise to the final angular scattering data. To match our experimental setup, we simulate FPM to compute the complex field scattered by the sample. As noted earlier, we use FTLS to obtain the angular scattering.

**Fig. 2:**
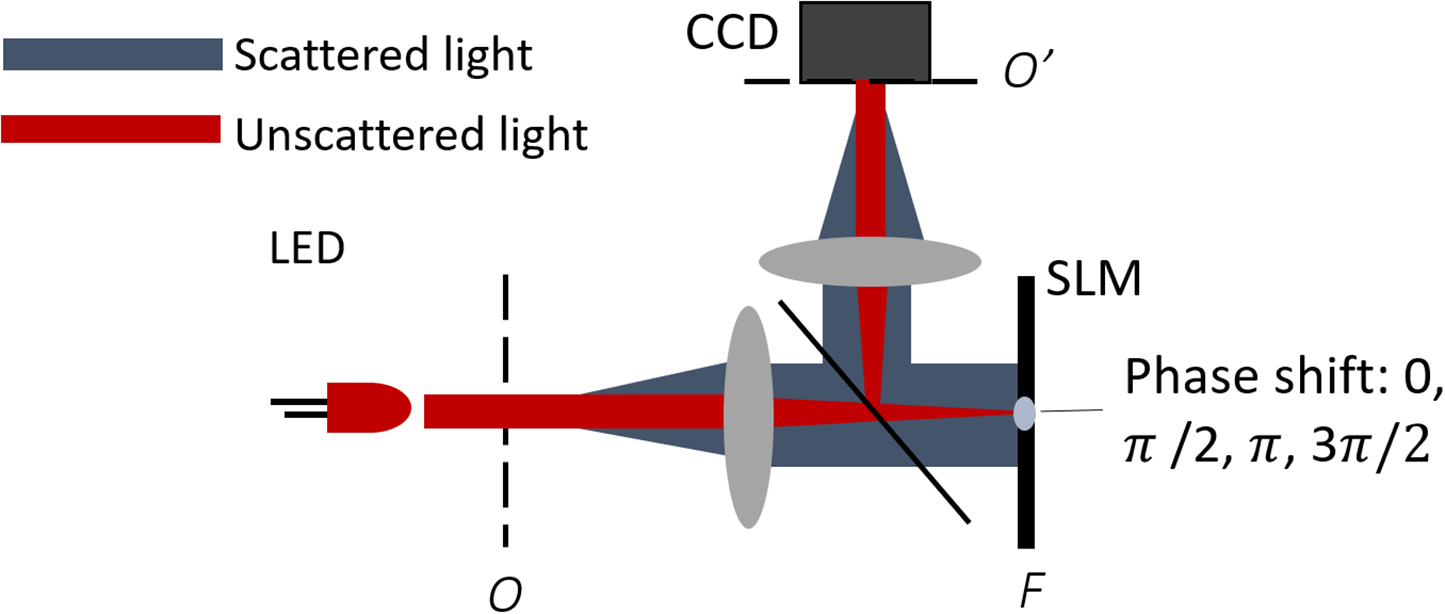
Conceptual diagram of a Fourier phase microscope; the light scattered by the sample (O) is imaged to the CCD array ($O^{\prime }$) and the unscattered light focuses to the center of a SLM at the Fourier plane (F), where four different phase shifts are applied.

FPM involves measuring four intensity interferograms. As shown in Fig. 2, an SLM located in a Fourier plane applies a phase shift of $\phi =\frac{m\pi }{2}$ for m=0,1,2,3 to a small central spot where the unscattered light hits the SLM. At the image plane $O^{\prime }$, this creates the m’th phase-shifted reference wave $E_{r}$, which interferes with the field scattered by the sample, or $E_{s}$. The corresponding four interferograms are

$$I_{m}(x,y)={\left|E_{r}\right|}^{2}+{\left|E_{s}(x,y)\right|}^{2}+2|E_{r}||E_{s}(x,y)|\cos(\phi_{s}(x,y)+\frac{m\pi }{2})$$ (4)

for each phase shift. The amplitude of the sample beam is computed from the four measured intensity images from^27^

$$|E_{s}(x,y)|=\frac{1}{4}\sqrt{{\left(I_{3}-I_{1}\right)}^{2}+{\left(I_{0}-I_{2}\right)}^{2}},$$ (5)

when the plane wave $E_{r}$ is assumed to have unit amplitude for simplicity. The phase of the scattered field is similarly computed by

$$\phi_{s}(x,y)=\mathrm{atan}\;2(I_{3}-I_{1},I_{0}-I_{2}).$$ (6)

The complex field is then Fourier transformed to arrive at the angular scattering. The field’s amplitude is squared to compute the irradiance and azimuthally averaged to obtain I(θ).

### 2.5 Simulating Detector Noise

For a given input photon flux, the irradiance of the reference field and Mie theory-derived sample field are scaled as shown in Appendix A. The sample and reference fields are then added in complex field and squared to compute the irradiance. This is then converted to photoelectrons $e_{m}(x,y)$ by

$$e_{m}(x,y)=\eta \frac{I_{m}(x,y)A_{\mathrm{pix}}t_{\exp}}{E_{\text{photon}}},$$ (7)

where η is the quantum efficiency (a spectrally dependent parameter that describes the photon to electron conversion efficinency,) $I_{m}(x,y)$ is the irradiance in $W/m^{2}$, $A_{\mathrm{pix}}$ is the CCD pixel area, $t_{\exp}$ is the exposure time, and $E_{\text{photon}}=hc/\lambda$ is the photon energy. The detector noise is simulated by adding dark noise and shot noise to the raw intensity images.

Shot noise is due to the Poisson process of detection and has a Poisson distribution. For large numbers of photoelectrons, the noise is well approximated as additive white Gaussian noise with the variance $\sigma_{e}^{2}$ equaling the mean number of photoelectrons $\mu_{e}$. The contribution at each pixel due to shot noise is randomly sampled from $N(0,e_{m}(x,y))$, where $N(\mu ,\sigma^{2})$ is a random number sampled from a normal distribution with mean μ and variance $\sigma^{2}$. Finally, dark noise $\sigma_{d}$ must be added, and it depends on both the read noise $\sigma_{r}$ and the dark current $\mu_{I}$. It is also modeled as additive white Gaussian noise with variance

$$\sigma_{d}^{2}=\sigma_{r}^{2}+\mu_{I}t_{\exp},$$ (8)

and mean $\mu_{I}t$. These parameters were measured experimentally, as described in the next section. After adding shot noise and dark noise, the number of photoelectrons $e_{m}^{\prime }(x,y)$ at each pixel is given as

$$e_{m}^{\prime }(x,y)=e_{m}(x,y)+N(0,e_{m}(x,y))+N(\mu_{I}t,\sigma_{d}^{2}).$$ (9)

The final number of counts on the detector is given as

$$c_{m}(x,y)=\max(\text{round}\{Ke_{m}^{\prime }(x,y)+c_{0}\},2^{k-1}),$$ (10)

where K is the detector sensitivity, $c_{0}$ is the constant readout, and k is the number of bits in the detector. The max operation accounts for the potential saturation of the detector. The four images $c_{m}(x,y)$, which aside from noise are proportional to the irradiance I(x,y), are fed directly into Eqs. (5) and (6) to compute the noised amplitude (to within a scalar constant) and phase of the scattered field, respectively. The camera parameters in Table 2 were measured experimentally as described by the EMVA Linear Standard^28^ and in Appendix B. The quantum efficiency is the only parameter that cannot be easily measured, and it was taken from the camera specifications.

**Table 2:** Parameters of Andor Luca CCD camera.

Readout	$c_{0}$	504 counts
Sensitivity	K	0.59
Quantum efficiency	η	0.23
Read noise	$\sigma_{r}$	14.1 electrons
Dark current	$\mu_{I}$	$0.44\;{\text{electrons}}^{2}/s$

### 2.6 Fitting Algorithm

The forward model for computing angular scattering from a number distribution of scatterers, n, is

I=Tn, (11)

where I is a column vector of the scattered intensity versus angle (I(θ)) and T is a matrix with columns that are the scattered intensity as a function of angle for scatterer diameters between $d_{\min}$ and $d_{\max}$, as computed by Mie theory. In this work, the minimum and maximum scatterer diameters simulated were 0.01 and 4  μm, and steps were taken every 10 nm. The inverse problem is to solve for the number density n, given a measurement of I, and the known Mie theory table T. This is known to be an ill-conditioned inverse problem, meaning small variations in the noise on I are magnified to cause large changes in the estimate of n.^29^^,^^30^

To solve this ill-conditioned inverse problem, *a priori* information must be introduced. A common approach is to assume a functional form for the number density n, often a log normal for particle size distributions.^29^ The log normal size distribution as a function of diameter d and input variables mean μ and standard deviation σ is defined as

$$n(d;\mu ,\sigma )=\frac{1}{dS\sqrt{2\pi }}\;\exp(-\frac{{\left(\ln\;d-M\right)}^{2}}{2S^{2}}),$$ (12)

for $d_{\min}\leq d\leq d_{\max}$, where

$$M=\ln(\frac{\mu^{2}}{\sqrt{\mu^{2}+\sigma^{2}}})\;\text{and}\;S=\sqrt{\ln(1+{\left(\frac{\sigma }{\mu }\right)}^{2})}.$$ (13)

The optimization problem is then to choose the (μ,σ) values that minimize the $\ell_{2}$ norm of the $\chi^{2}$ error between the scattering I(θ) from Eq. (11) and the measured $I_{d}(\theta )$, or

(μ^,σ^)=arg minμ>0,σ>0,‖Itheory(μ,σ)−IdS2‖22, (14)

where S is a diagonal matrix containing the elements

$$s_{i}=\sqrt{\frac{\underset{\phi }{\mathrm{var}}\{I(\theta_{i},\phi )\}}{p(\theta_{i})}},$$ (15)

which are the azimuthal standard deviations of $I(\theta_{i},\phi )$ normalized by the square root of the number of pixels $p(\theta_{i})$ in each annular bin. The $p(\theta_{i})$ weighting de-emphasizes the annular bins with the fewest pixels over which to average. Equation (14) is solved using Matlab’s built in fmincon optimizer.

Because we are not estimating the number of scatterers present and detector noise has the effect of adding an offset to the 1D angular scattering (as described in more detail in Sec. 4), the fitting procedure allows for an arbitrary scaling and offset factor between the theory and data, or

$$I_{\text{theory}}=[\begin{array}{cc} (Tn{)}_{1} & 1\\ (Tn{)}_{2} & 1\\ . & .\\ . & .\\ . & . \end{array}][\begin{array}{c} c_{1}\\ c_{0} \end{array}],$$ (16)

with the constant offset $c_{0}$ and scaling factor $c_{1}$. These values are determined during each iteration of the optimization algorithm for a given (μ,σ) pair by finding the least squares fit between the data and scaled theory.

Note that the forward model both samples from a smooth, analytic size distribution and does not attempt to solve for the locations of the scatterers. Sparse sampling of size distributions, interference between scatterers, and detector noise all introduce uncertainty into size distribution parameter estimates. The next section describes how we quantify the effect of each of these noise sources and compare them to understand what limits the accuracy of size estimates.

### 2.7 Quantifying the Effect of Noise on Size Estimates

To quantify the noise added by sampling, interference, or detector processes, many simulations are run while changing a parameter, such as the number of scatterers, their locations, or the random detector noise. The collection of corresponding angular scattering plots gives a sense of how that noise source is carried through to the angular scattering. The signal-to-noise ratio (SNR) at each scattering angle is computed by dividing the mean value across all simulations by the standard deviation, or

$$\mathrm{SNR}(\theta )=\frac{{\text{mean}}_{i}(I(\theta ,i))}{{\mathrm{std}}_{i}(I(\theta ,i))},$$ (17)

where I(θ,i) is the angular scattering from the i’th run of the simulation. A mean SNR value $\bar{\mathrm{SNR}}$ across all angles is computed as

$$\bar{\mathrm{SNR}}={\mathrm{mean}}_{\theta }(\mathrm{SNR}(\theta )).$$ (18)

Although one use of simulations is to understand how various noise sources contribute to noise in the angular domain, it is important to take this a step further to consider how it propagates through the fitting process and affects size estimates. This result depends significantly on the algorithm used to estimate size distributions. In this paper, we used a “best fit” approach that models the simulated size distributions with a single log normal distribution, as described in the previous section. Other approaches to solving the inverse problem are briefly noted in Secs. 4 and 5.

To quantify the error in estimated size distributions, we defined a fractional error in the estimated mean of the scatterer ensemble as

$$f_{\mu }=\frac{\left|\mu_{\mathrm{est}}-\mu_{\mathrm{samp}}\right|}{\mu_{\mathrm{samp}}},$$ (19)

and similarly a fractional error for the standard deviation as

$$f_{\sigma }=\frac{\left|\sigma_{\mathrm{est}}-\sigma_{\mathrm{samp}}\right|}{\sigma_{\mathrm{samp}}},$$ (20)

where the parameters $\mu_{\mathrm{samp}}$ and $\sigma_{\mathrm{samp}}$ are the mean and standard deviation of the scatterer sizes generated using the inversion sampling method previously described, respectively. The estimated parameters indicated by subscript “est” are the mean and standard deviation of the estimated size distribution over the range $d_{\min}\leq d\leq d_{\max}$. The mean $\bar{\mathrm{SNR}}$ and the fractional error in size estimates are useful parameters for comparing the effects of each noise type. A slightly modified $\bar{\mathrm{SNR}}$ definition is used for detector noise and is described in a later section.

## 3 Simulation Results

### 3.1 Noise Sources

**Fig. 3:**
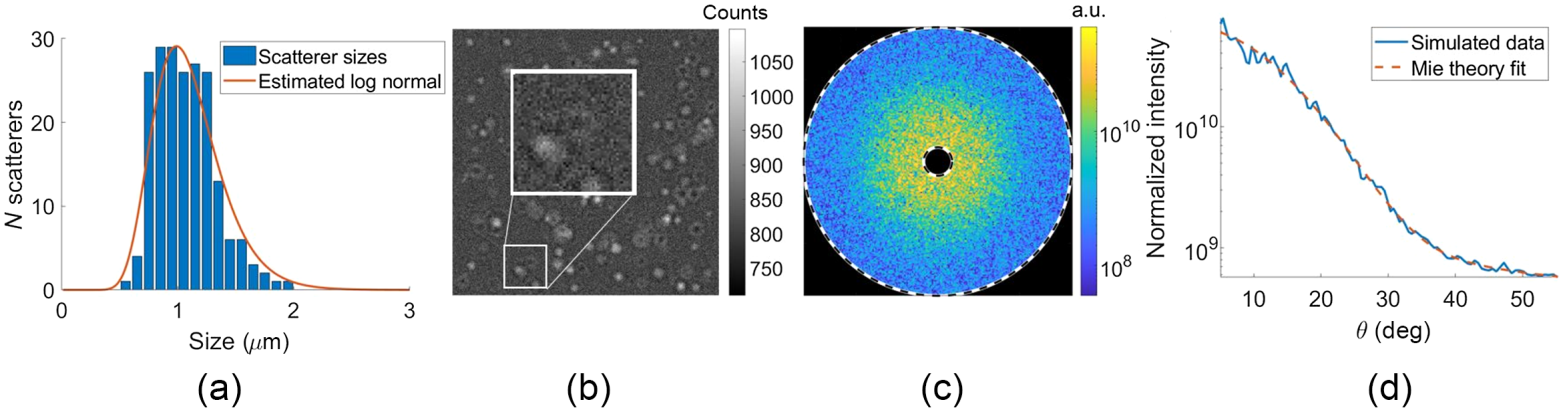
(a) Histogram of scatterer diameters from randomly sampling a μ=1.1  μm, σ=300  nm log normal with 200 scatterers, and estimated log normal corresponding to Mie theory fit. (b) Corresponding intensity image of the scatterers placed at different (x,y,z) locations, with detector noise added. Field of view is 40  μm on a side. (c) 2D scattering intensity after Fourier transform of the complex field; inner/outer dashed lines indicate 5/55 deg. (d) 1D scattering (blue, solid) from azimuthally averaging the 2D scattering and Mie theory fit (orange, dashed).

An example of a cell’s simulated scattering and its fit is shown in Fig. 3. The histogram in Fig. 3(a) shows the scatterer diameters from sampling a μ=1.1  μm, σ=300  nm log normal with 200 scatterers. These are then randomly placed in a 40×40×5  μm region and used to generate the four intensity images measured by the camera under different phase shifts. Figure 3(b) shows the zero phase-shift intensity image of the sample with the detector noise added (visible in enlarged inset). The sample’s complex field computed from Eqs. (5) and (6) is Fourier transformed to arrive at the 2D scattered intensity plot in Fig. 3(c), where the inner dashed line is polar angle of 5 deg and the outer dashed line is 55 deg. Finally, this 2D scattering is azimuthally averaged to arrive at the 1D angular scattering in Fig. 3(d), which is shown with its Mie theory fit of μ=1.09  μm and σ=250  nm. The log normal size distribution corresponding to this fit is plotted with the histogram in Fig. 3(a).

The remainder of Sec. 3.1 establishes the relationships between simulation parameters (number of scatterers, log normal width σ, cell size, and exposure time) and their effects upon angular scattering by individual noise sources (sampling, interference, and detector noise) in isolation. Section 3.2 then considers the three noise sources acting in combination and the resulting effect upon the accuracy of size distribution parameter estimates.

#### 3.1.1 Sampling noise

**Fig. 4:**
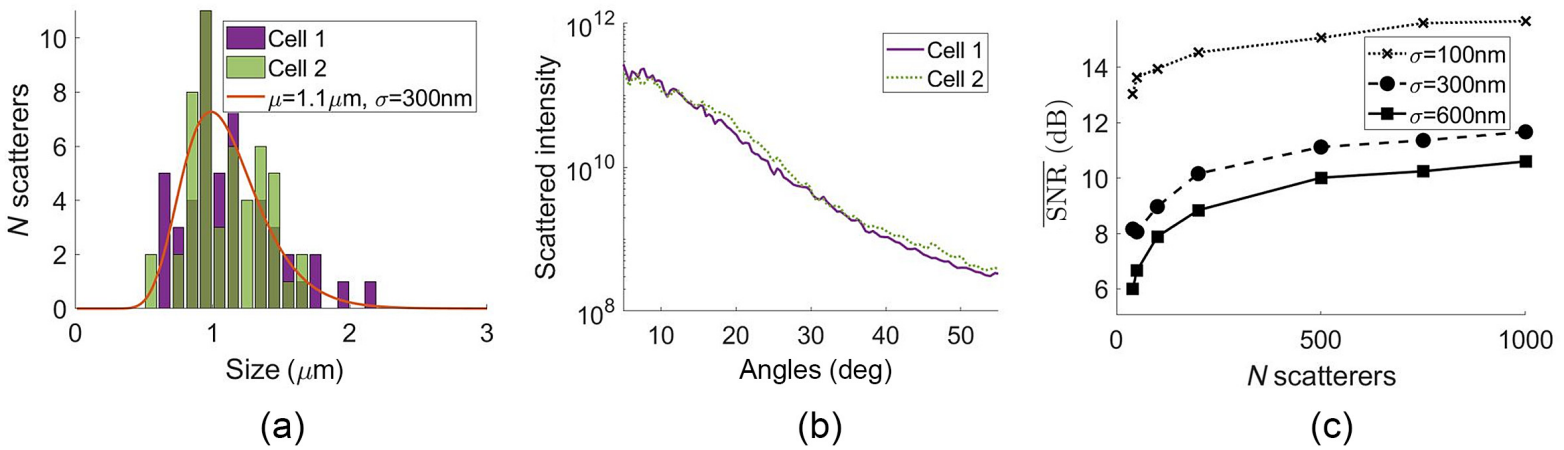
(a) Two realizations of sampling the same μ=1.1  μm, σ=300  nm size distribution with 50 scatterers, and (b) the corresponding angular scattering without varying the scatterer locations or detector noise. (c) $\bar{\mathrm{SNR}}$ dependence on number of scatterers for μ=1.1  μm and σ=100, 300, and 600 nm.

To demonstrate the meaning of sampling noise, simulations were run while only changing the sizes of the scatterers. Figure 4(a) shows an example of two histograms generated by randomly sampling a log normal curve with μ=1.1  μm and σ=300  nm and distributing 50 scatterers using the procedure described in Sec. 2.2. Despite being drawn from the same log normal, these two sampled distributions have different angular scattering, shown unnormalized in Fig. 4(b). This variation due to sampling of the size distribition is the “sampling noise.” Sampling noise decreases as the number of scatterers increases, and as the distribution width approaches zero. To quantify these trends, we ran 100 simulations while varying only scatterer sizes to compute the mean SNR over all angles as defined in Eq. (18). We repeated this for cells with between 50 and 1000 scatterers and for distribution widths of 100, 300, and 600 nm, yielding the plots in Fig. 4(c). As expected, the $\overset{¯}{\mathrm{SNR}}$ increases as the number of scatterers increases and as σ decreases.

#### 3.1.2 Interference noise

To study the influence of interference noise, the scattering is compared for an ensemble of scatterer sizes in which only their locations are changed and not their sizes or the detector noise. The variations in the angular scattering are then due to the changes in interference between scatterers.

**Fig. 5:**
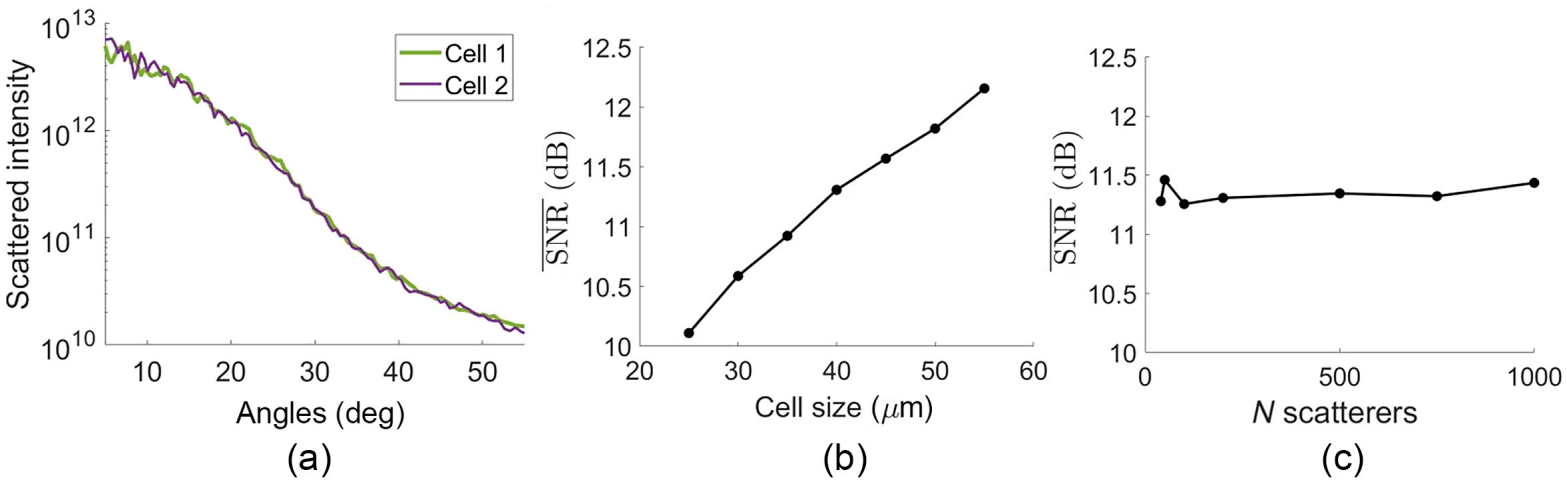
(a) Two realizations of interference noise from changing the positions of 200 scatterers within a 40×40×5  μm cell. (b) $\bar{\mathrm{SNR}}$ computed over 100 simulations of randomizing 200 scatterer locations for a μ=1.1  μm, σ=300  nm distribution for cell widths between 25 and 55  μm. (c) $\bar{\mathrm{SNR}}$ computed over 100 simulations of randomizing the locations of between 40 and 1000 scatterers for a μ=1.1  μm, σ=300  nm distribution, and for a cell width of 40  μm.

Figure 5(a) shows two examples of scattering in which only the scatterer locations are changed for a 40×40×5  μm cell with 200 scatterers. The difference between the two plots is due to the high frequency changes in the interference pattern. The $\overset{¯}{\mathrm{SNR}}$ is again computed over 100 simulation runs to quantify the effect of interference noise. The results are shown in Fig. 5(b) for changing the D×D×5  μm cell size for between D=25 and 55  μm and in Fig. 5(c) for changing the number of scatterers between 40 and 1000 for D=40  μm.

#### 3.1.3 Detector noise

##### Bead calibration

**Fig. 6:**
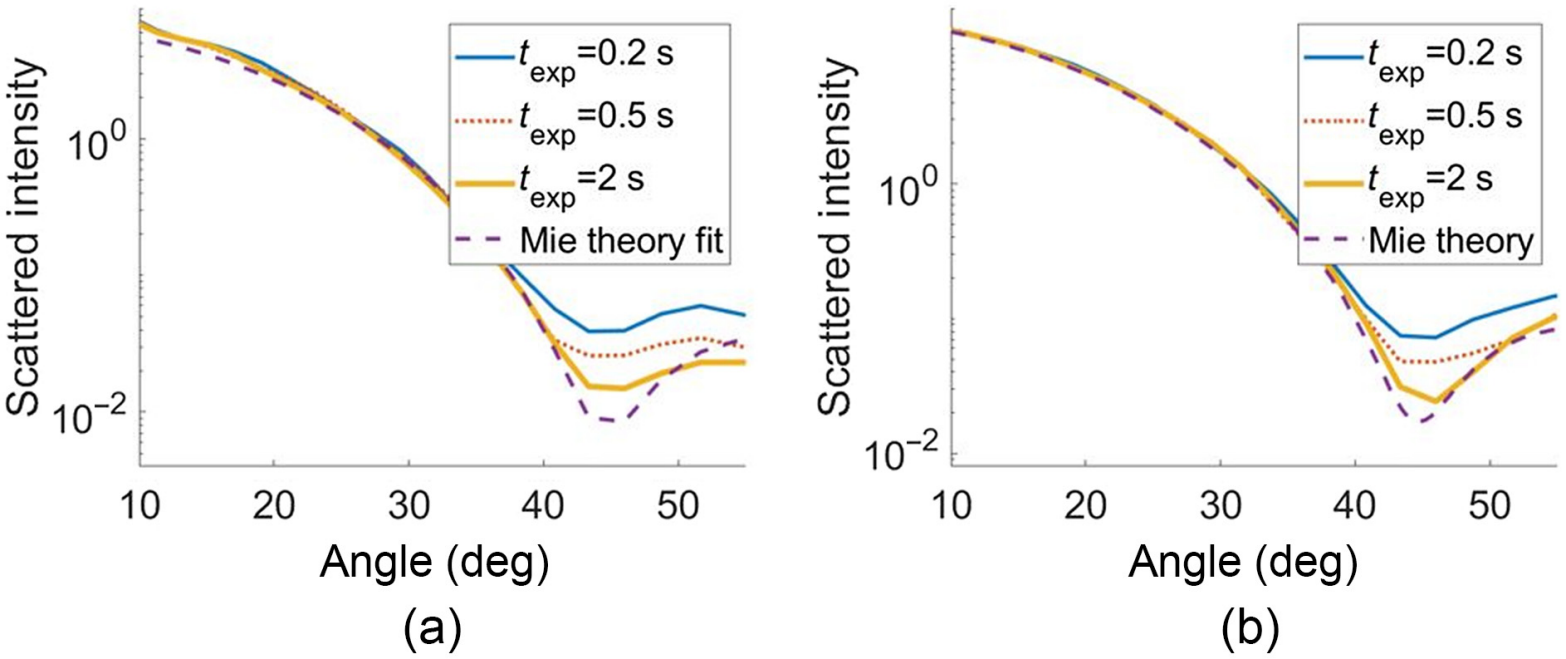
(a) Experimental angular scattering for bead immersed in glycerol at exposure times of 0.2 (blue solid), 0.5 (orange dotted), and 2 s (yellow thick) with a Mie theory fit (purple dashed) showing a diameter estimate of 0.972  μm. (b) Simulated angular scattering from a single 0.972  μm bead immersed in glycerol for the same exposure times and without detector noise.

To validate our simulation tool’s ability to accurately model detector noise, we first compared experimental and simulated scattering at several exposure times for a bead immersed in glycerol. This sample has signal levels close to a mitochondrion’s scattering in a cell due to the comparable size and refractive index contrast. As shown in Fig. 6(a), a Mie theory fit to the $t_{\exp}=2\;s$ experimental data yielded a size estimate of 0.972±0.039  μm, where the uncertainty is where the $\chi^{2}$ error metric doubles. The $t_{\exp}=2\;s$ data were chosen for fitting, so the effect of the detector noise on the fit accuracy is minimized, making the data closest to an ideal Mie curve.

##### Noise effects

To demonstrate the difference between the detector noise-free Mie theory fit and the experimental measurements, the fit in this example did not allow for the offset $c_{0}$ described in Eq. (16); had this been allowed, the three curves at different times would have collapsed onto each other. By varying the exposure time both in experiments and in simulations, we can see changes in the scattering signal that are due to detector noise. The photon flux in the simulations was set to 14,000 photons/pixel/s to match the LED’s irradiance on the day of measurement, as was measured by plotting the average number of counts in a bead-free region of the sample versus exposure time. The data in Fig. 6 are normalized by the exposure time, so the shape of the scattering can be compared. The experimental and simulated results in Figs. 6(a) and 6(b) show the same effect of the noise floor being raised as exposure time decreases due to the presence of additional shot noise. On the semi-log scale, this effect is most evident between 40 deg and 55 deg. This gives us confidence that the simulation of FPM is inducing detector noise effects consistent with the experimental measurements. We later show with simulations of scatterer ensembles that this detector noise effect contains a large DC “offset” term in the angular domain, which requires the use of the offset $c_{0}$ to accurately fit scatterer ensembles.

**Fig. 7:**
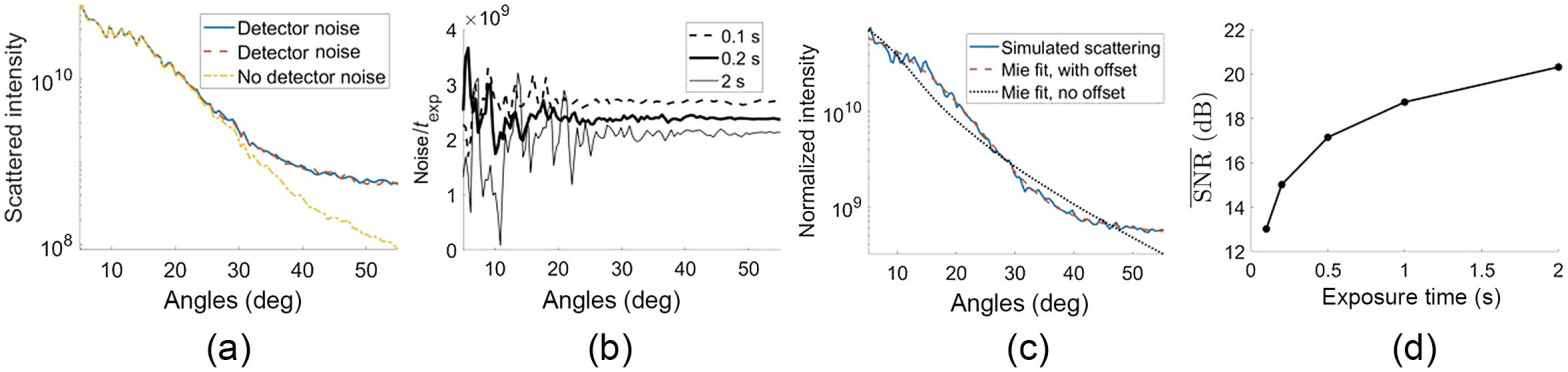
(a) Scattering from two realizations of detector noise and without detector noise. (b) Difference between noised and unnoised signal scaled by the exposure times. (c) Example fits to simulated scattering with and without allowing for constant offset in the fit. (d) $\bar{\mathrm{SNR}}$ [as defined in Eqs. (22) and (18)] versus exposure time.

To demonstrate the effect of detector noise on cell simulations, an example cell was simulated multiple times using the exact same scatterers and locations while only varying the detector noise. The simulated photon flux was set here to 30,000 photons/pixel/s to match the typical irradiance of the LED. A “detector-noise-free” version of the scattering was also generated for comparison, where detector noise was not added to the raw interferograms. Figure 7(a) shows the angular scattering intensity with two realizations of detector noise on the same cell, and the “unnoised” data (i.e., without adding detector noise). In Fig. 7(b), we show on a linear scale the difference between the noised and unnoised plots scaled by the exposure time, or

$$\frac{\text{noise}(\theta )}{t_{\exp}}=\frac{I(\theta )-I_{\mathrm{no}-\det-\text{noise}}(\theta )}{t_{\exp}},$$ (21)

where I(θ) includes the detector noise and $I_{\mathrm{no}-\det-\text{noise}}(\theta )$ does not. This noise can be expressed as the sum of a constant DC term and an angle-varying AC term. Note that when scaled by the exposure time, the AC term increases with exposure time (best seen at the noisier low angles, where fewer pixels are averaged over), whereas the DC term decreases with exposure time (best seen at the high angles, where more pixels are averaged over). The presence of a DC term in the detector noise suggests the need for a DC offset term in the fitting procedure. To demonstrate the importance of allowing for an offset during the fitting procedure in the presence of detector noise, Fig. 7(c) shows an example cell with a fit both with and without the offset. Note that the Mie fit only resembles the angular scattering when the offset term is included in the fitting procedure. Because the DC component of the detector noise does not affect the fit when the offset is allowed, it should not be included as a noise term when computing the SNR. Furthermore, because the DC component of the noise remains the same for different realizations of detector noise with the same exposure time, the $\bar{\mathrm{SNR}}$ definition must be modified so that it is not erroneously included as part of the signal. We therefore define the detector-noise-related SNR(θ) as

$$\mathrm{SNR}(\theta )=\frac{{\text{mean}}_{i}(I_{\mathrm{no}-\det-\text{noise}}(\theta ,i))}{{\mathrm{std}}_{i}(I(\theta ,i)-I_{\mathrm{no}-\det-\text{noise}}(\theta ,i))},$$ (22)

so the detector noise-induced constant offset is not counted as part of the signal. The angle-averaged metric $\bar{\mathrm{SNR}}$ is still computed using Eq. (18). The $\bar{\mathrm{SNR}}$ is plotted for exposure times between 0.1 and 2 s, which corresponds to between 3000 and 60,000 photons/pixel.

### 3.2 Effect of Noise Sources on Size Estimates

**Fig. 8:**
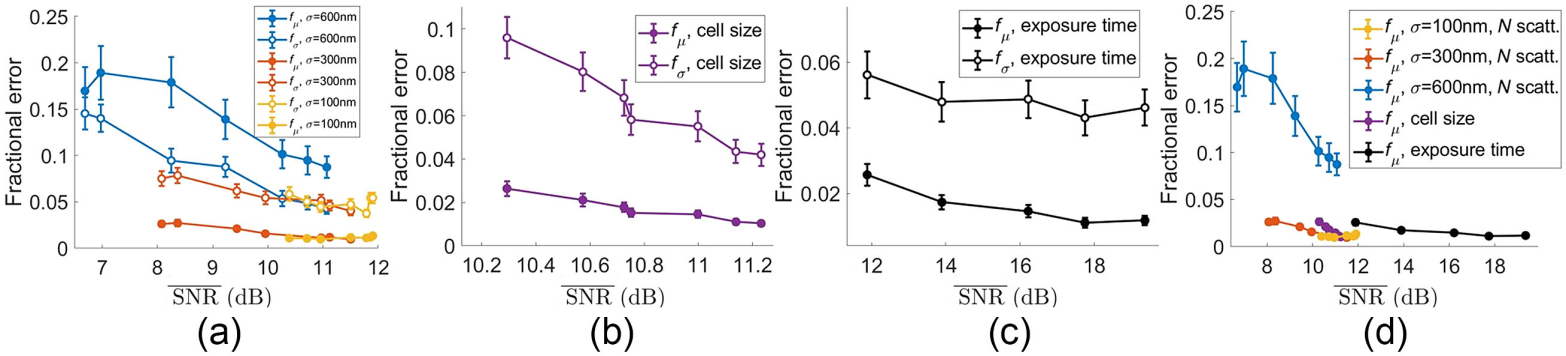
Comparison of fractional error in μ (filled markers) and σ (open markers) versus mean SNR for (a) varying number of scatterers varied between 40 and 1000 and varying size distribution width σ=100, 300, and 600 nm, and for fixed parameters μ=1.1  μm, D=55  μm, and $t_{\exp}=2\;s$. (b) Varying the cell’s lateral dimensions between 25 and 55  μm and with fixed parameters μ=1.1  μm, σ=300  nm, 750 scatterers, and $t_{\exp}=2\;s$. (c) Varying $t_{\exp}$ between 0.1 and 2 s and fixed parameters μ=1.1  μm, σ=300  nm, 750 scatterers, and D=55  μm. (d) Compiled $f_{\mu }$ results from (a)–(c) plotted on the same axes for comparison. Error bars (some smaller than markers) indicate standard error of the mean over 100 simulations and fits.

To study the impact of sampling, detector, and interference noise on the accuracy of size estimates, we now include all three noise types in the simulated data, so the noise characteristics are representative of experimental cell scattering. We rely on the dependence we have established between the simulation parameters and the $\bar{\mathrm{SNR}}$ in the results shown in Figs. 4–7, namely that distribution width σ and number of scatterers N control the level of sampling noise, cell size D controls the interference noise, and $t_{\exp}$ controls the detector noise. To explore the effect of noise sources on size estimates, the level of only one noise source was changed at a time by changing the relevant experimental parameter, and 100 simulations were run to generate each datapoint. Although the other noise sources were also present, the levels of those noise sources were held constant. In Fig. 8(a), the level of sampling noise was varied by changing the distribution width σ and the number of scatterers N to see how the ensuing noise variations affected the size estimates. In Fig. 8(b), the level of interference noise was varied by changing the cell size. The level of detector noise was held constant by holding the field of view fixed at 55×55  μm while the cell size was varied. In Fig. 8(c), the level of detector noise was varied by changing the exposure time. For all of these results, the fractional error in size estimates tends to decrease as the SNR increases. Finally, Fig. 8(d) combines the $f_{\mu }$ results from Figs. 8(a)–8(c) on the same axes for comparison.

## 4 Discussion

### 4.1 Noise Sources

Each of the noise sources investigated manifests itself differently in the angular domain. The characteristics of the noise sources determine the degree to which each noise source affects the Mie theory fit.

#### 4.1.1 Sampling noise

Sampling error is characterized by slowly varying changes in the angular scattering distribution. The two example histograms in Fig. 4(a) result in the two scattering intensities in Fig. 4(b). Note that there are regions of the angular range where cell 2’s scattering is consistently higher than cell 1’s, such as from 15 deg to 25 deg and from 37 to 50 deg. This indicates a slowly varying noise component relative to the high frequency ripples also visible in the data (which are mostly from interference noise). The noise-free Mie theory-based angular scattering used to fit the data tends to be a smooth, monotonic signal for broad distributions of organelle-sized scatterers. Therefore, one would expect these low-frequency variations due to sampling noise to affect the Mie theory fits substantially, as discussed with the fit results.

Before looking at fit stability, we look at the $\bar{\mathrm{SNR}}$ metric to quantify the impact of number of scatterers N and distribution width σ. It is intuitive that the moments of the sampled distribution become closer to the simulated distribution as the number of scatterers N increases (i.e., law of large numbers). This also means that different realizations of sampling the same population are more consistent for larger N, resulting in less variation in the angular scattering signal and therefore higher sampling-related $\bar{\mathrm{SNR}}$. This is seen in the increasing $\bar{\mathrm{SNR}}$ with N in Fig. 4(b). Notably, this trend is most significant between 40 and 200 scatterers. There is a larger improvement in $\bar{\mathrm{SNR}}$ between 40 and 200 scatterers than from 200 to 1000 scatterers. This may have implications for which types of single cells are least amenable to robust estimates of organelle size distribution parameters via angular scattering. In addition, having a narrower distribution means that the $\bar{\mathrm{SNR}}$ is greater than 12 dB even for N=40. The curves of $\bar{\mathrm{SNR}}$ versus the number of scatterers are shown for widths of 100, 300, and 600 nm in Fig. 4(c) to demonstrate that the $\bar{\mathrm{SNR}}$ is higher for narrower distributions.

#### 4.1.2 Interference noise

For interference noise, $\bar{\mathrm{SNR}}$ is strongly affected by the cell size. This is because the farthest separation between scatterers in the spatial domain determines the highest spatial frequency of interference fringes that can occur in the angular domain. Larger cell dimensions have greater maximum scatterer separations and smaller interference features. Because finer (high spatial frequency in the 2D angular scattering) interference fringes are averaged out more than coarser features, the large cell sizes improved $\bar{\mathrm{SNR}}$. This result is visible in Fig. 5(b), where $\bar{\mathrm{SNR}}$ increases with cell size parameter D. Interestingly, increasing the number of scatterers while keeping the cell size the same does not affect the $\bar{\mathrm{SNR}}$ plot in Fig. 5(c), implying that the density of scatterers or average separation between scatterers does not determine the spatial size of interference noise. We note that this simulation platform assumes spatially coherent illumination across the cell. The effect of cell size on interference noise would be different if there were finite spatial coherence because the spatial coherence area would limit the maximum distance at which organelles could interfere.

#### 4.1.3 Detector noise

Without doing a detailed analysis of noise propagation through Eqs. (5) and (6), it is nevertheless possible to gain insight into how detector noise influences angular scattering, as shown in Fig. 7. Most notably, the constant offset component shown in Fig. 7(b) that decreases with exposure time can be explained by the noise power spectrum of white noise. For an image with independent, identically distributed noise (i.e., independent pixels, each with the same probability distribution), the ensemble average of the Fourier transform’s modulus squared is constant. Because the reference plane wave dominates the overall signal in all of the raw intensity images, it is a good approximation that the detector noise in those intensity images is white noise. When azimuthally averaged, this noise therefore contributes a DC component in the angular domain that scales approximately as the square root of exposure time. Because the Mie scattering itself increases linearly with time, the relative contribution of the noise-induced DC component increases as exposure time decreases, in agreement with Fig. 7(b) as time decreases from 2.0 to 0.2 s. The white noise also contributes AC components to the angular scattering, idiosyncratic to the particular distribution of signal in the four intensity images.

Although these simulations provide insight into the nature of the exposure time-dependent offset in the angular domain, it is also apparent that this noise has a minimal effect on the ability to fit size distributions for the range of exposure times explored in this paper. Figure 8(c) shows that the metric $f_{\mu }$ remains below 3% error for all values of exposure time explored, and the value of $f_{\sigma }$ is below 6%. Furthermore, it should always be possible experimentally to increase the source brightness or exposure time to an acceptable level, so shot noise is not the limiting factor in estimating size distributions. In particular, the light levels used in the simulations corresponded to a low-power LED, whereas a superluminescent diode would have higher irradiance in the field of view.

### 4.2 Effect of Noise on Size Estimates

Although we have described the relationships that experimental and cell parameters have on $\bar{\mathrm{SNR}}$, this metric does not automatically map 1:1 to the accuracy with which the size distribution’s mean μ and width σ are estimated. Although the error metric generally decreases as $\bar{\mathrm{SNR}}$ increases, there is not a fixed relationship between the $\bar{\mathrm{SNR}}$ and size estimate error. This means that some of the plots in Fig. 8(d) have different error metric values despite overlapping in their $\bar{\mathrm{SNR}}$ range. The slopes also differ between the plots; notably the blue plot $f_{\mu }$ for changing the number of scatterers in a distribution with σ=600  nm drops sharply from 20% error to <10% error between $\overset{¯}{\mathrm{SNR}}=7$ and 10 dB, whereas the black plot from increasing the exposure time only changes by <3% over more than 6 dB. Increasing the cell size (and thereby reducing interference noise) also has a notably steep slope (purple plot), although it covers <2  dB of $\bar{\mathrm{SNR}}$ values.

There are several other reasons for the differences in error metric values and slope. Factors such as the shape of the angular scattering curve also influence the stability of solving the inverse problem and therefore of estimating size distributions. For example, the mean of the widest distribution with σ=600  nm has a higher error than for σ=300  nm, despite the overlap in the $\bar{\mathrm{SNR}}$ range. This is likely due to the fact that wider distributions have a more featureless angular scattering shape because any minima or other features are averaged over a wide range of angular locations from different scatterer sizes (e.g., compare the minimum in the single 0.972  μm scatterer in Fig. 6 with the monotonically decreasing angular scattering plot in Fig. 3(d) from a log-normal distribution). These monotonically decreasing (aside from the high frequency interference noise) scattering curves have fewer features, making the fitting process more susceptible to noise. There is also no notable difference in fitting performance between the 100 and 300 nm wide distributions, despite the difference in $\bar{\mathrm{SNR}}$. This is because interference noise begins to limit the fitting accuracy when the sampling noise is low enough.

In addition, each type of noise source has a different behavior in the angular domain. The $\bar{\mathrm{SNR}}$ metric is an averaged parameter that obscures the angular dependence of the SNR. For example, the azimuthal averaging reduces the relative effects of interference and the AC component of the detector noise at the highest scattering angles where there are more pixels in an annular bin. However, the sampling error is an azimuthally symmetric effect that does not benefit from averaging over more pixels. In addition, the spatial frequency content of the error in the 1D azimuthal plot plays an important role. For example, the slowly varying sampling noise has a large effect on the Mie theory fits (and thus the error metrics $f_{\mu }$ and $f_{\sigma }$) because the Mie scattering has similar spatial frequencies in the angular domain. The high spatial frequency fluctuations of the interference noise, however, affect the fit accuracy less, for example, as seen in the fit through the data in Fig. 3(d).

Overall, the sampling noise causes the highest $f_{\mu }$ and $f_{\sigma }$ values when σ=600  nm. The $f_{\mu }$ values are larger than 10% for this widest log normal distribution and are smaller than 5% for all other sets of simulation parameters. This suggests that the distribution width of a cell’s scatterers is significant in determining whether this fitting approach is capable of estimating organelle sizes accurately. For such wide distributions, a large number of scatterers (more than 750) is need to bring $f_{\mu }$ to below 10%. This indicates that the scattering from a finite number of scatterers with widely distributed sizes is poorly modeled by a continuous size distribution. This sampling “error” is equivalent to an inaccurate assumption of the distribution’s functional form, meaning that it could potentially be addressed by different approaches to the inverse problem.

## 5 Conclusion

To our knowledge, this is the first effort to characterize how accurately angularly resolved light scattering can size organelle populations representative of a single cell’s contents. We used simulations to investigate the experimental and cell parameters that influence various noise types and then studied how the levels of each noise source influence the size-distribution estimation accuracy. The results provide insight into the properties of sampling, interference, and detector noise, as well as their impact upon angular scattering fits. Although the detector noise is specific to the FPM and FTLS methods of data collection, the interference noise and sampling noise are generalizable to any angular scattering method because they are properties of the cell rather than the measurement system. We chose ranges of simulation parameters to represent experimental conditions and cell properties as closely as possible, including experimental measurements of our detector properties. Although the simulations presented in this work assumed full spatial coherence, a future version could include variables to model partial spatial coherence. This would capture the effects of interference noise more accurately because scatterers separated by more than the spatial coherence length at the sample would not coherently interfere.

We found that, for σ≈300  nm, the fractional errors due to sampling, interference, and detector noise are comparable and remain below 5% error in the $f_{\mu }$ [c.f. Fig. 8(d)], with as few as 40 scatterers. For wider size distributions (σ≈600  nm), the sampling noise dominates the error in size estimates and requires more than 750 scatterers to estimate the mean with <10% error [c.f. Fig. 8(a)]. One important implication of this result is that there is little to gain from reducing detector or interference noise in regions of parameter space where the fitting accuracy is dominated by the sampling error. This motivates using inversion approaches that do not require assuming the functional form of size distributions; this is an approach that we plan to explore in future work.

It is also worth considering the simplifications in this simulation work that are not representative of experimental cell scattering. For example, this work assumed an idealized cell model with a homogeneous and identical refractive index for all scatterers, no nucleus-sized scatterers, and all spherical scatterers. In addition, the results indicate that the log-normal assumption might be the limiting factor in estimating size distributions accurately, especially for distributions with widths at or beyond 600 nm. This suggests that it is worth investigating the validity of these assumptions for single cells. We are currently using tomographic refractive index maps of single cells to investigate the agreement between these model simplifications and real cells. Although spheroids are known to scatter on average similar to their area equivalent spheres,^31^ future work could also include a more sophisticated simulation model to model spheroidal scatterers, for example, using T-matrix theory.^32^^,^^33^

## 6 Appendix A: Scaling Reference Beam Strength in Simulation

To simulate the four interferograms, the strength of the scattered field must be scaled relative to the incident plane wave’s strength. Given an input photon flux corresponding to an incident plane wave with irradiance $I_{0}$, the radiant flux $\Phi_{s}$ scattered by a microscopic object is

$$\Phi_{s}=I_{0}C_{\mathrm{sca}},$$ (23)

where $C_{\mathrm{sca}}$ is the scattering cross section in units of meters squared. For a dielectric sphere, a scattering cross section is computed by Mie theory^34^ over the solid angle subtended by a microscope objective as

$$C_{\mathrm{sca}}=\int_{0}^{2\pi }\int_{0}^{\theta_{\max}}\frac{|\chi {|}^{2}}{k^{2}}\;\sin(\theta )d\theta \;d\phi ,$$ (24)

where $\frac{|\chi {|}^{2}}{k^{2}}$ is the differential scattering cross section. ^34^

With $\Phi_{s}$ thus defined via Eq. (23), the average irradiance of the entire field of view is then

$$I_{s}=\frac{\Phi_{s}}{A_{\mathrm{obj}}},$$ (25)

where $A_{\mathrm{obj}}$ is the detector field of view demagnified through the imaging system to the object plane. The scattered field $E_{s}(x,y)$ computed by Mie theory is then scaled such that

$$\frac{\underset{x,y}{\sum }{\left|E_{s}(x,y)\right|}^{2}}{N_{\text{pixels}}}=I_{s},$$ (26)

meaning that the average irradiance of the scattered light is $I_{s}$. The interferograms are then computed by summing the sample field and the reference field for each phase shift and taking the modulus squared, which is equivalent to Eq. (4).

## 7 Appendix B: Characterizing Detector Noise

Detector noise is specific to the detector used because the quantum efficiency, sensitivity, and readout noise are dependent on the individual detector’s performance. A series of simple experiments can be used to characterize detector performance because not all parameters are provided on specification sheets. Our experimental Fourier phase microscope, described in further detail in Ref. 15, uses an Andor Luca DL-685M electron multiplying CCD-camera, operating without electron multiplication. To accurately simulate measurements of the camera with the correct levels of detector noise, it is important to know some basic properties of the camera performance, including dark noise, camera sensitivity, readout, read noise, and quantum efficiency. Quantum efficiency cannot be measured easily, so the value was taken from the specification sheet to be η=0.23 at the illumination wavelength of 780 nm.

The readout, read noise, dark current, camera sensitivity, and readout levels were measured experimentally as described in the EMVA Linear Standard.^28^ First, the readout $c_{0}$ was measured by taking the average pixel count for 100 dark frames, with the sensor covered and a short exposure time (1 ms) to minimize dark current buildup. The mean pixel value over 100 dark frames was $c_{0}=504$ counts, with a standard error of the mean of <1e−6 counts. Camera sensitivity K is the average number of counts $c_{m}$ that a pixel reads per incident photoelectron $e_{m}$. This relationship is defined as

$$c_{m}=Ke_{m}.$$ (27)

**Fig. 9:**
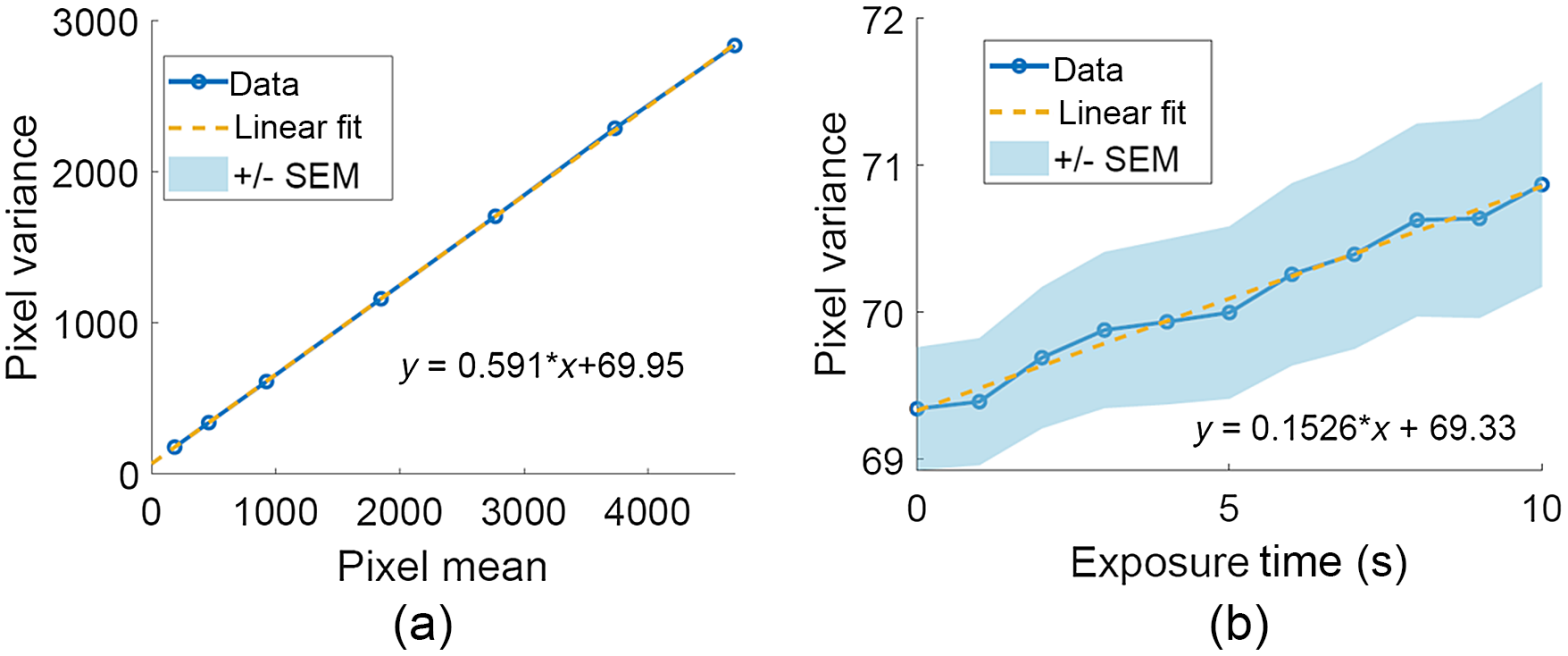
(a) Pixel variance versus pixel mean for exposure times between 0.1 and 5 s with the slope determining the camera sensitivity. Shaded error bars denote standard error of the mean but are smaller than the marker size. (b) Temporal pixel variance versus exposure time, with linear fit providing the read noise and dark current of the CCD. Error bars denote standard error of the mean.

Experimentally determining the sensitivity takes advantage of the relationship between the pixel mean and variance,^28^ or

$$\sigma_{c}^{2}=K^{2}\sigma_{d}^{2}+K(\mu_{c}-c_{0}),$$ (28)

where $\sigma_{c}^{2}$ is the pixel variance and $\mu_{c}$ is the pixel mean in counts.^28^ The sensitivity was measured by measuring 100 frames at exposure times between 0.1 and 5 s and computing each pixel’s variance across the 100 frames. For each exposure time, the mean pixel variance across the image was then plotted versus the mean pixel value with the readout subtracted off to only include the photo-induced signal. This plot is shown in Fig. 9(a). Using Eq. (29), the slope of this curve is the sensitivity K, which was found to be 0.59 counts/electron.

The read noise and dark current were computed by measuring 100 dark frames for a sequence of exposure times while the sensor was covered, so the only detected signal was dark noise. Hot pixels and cosmic rays were removed from consideration by thresholding the pixel values that were more than four standard deviations larger than the mean pixel value of each exposure time. This was found to reduce the standard error of the mean. For each exposure time, each pixel’s variance was computed across all frames and then averaged over the frame. A linear fit was then performed to a plot of the pixel variance versus exposure time as shown in Fig. 9(b). The shaded region denotes error bars of the standard error of the mean pixel variance. Equation (8) shows that dark noise scales linearly with exposure time, so the slope m of the line determines the dark current, or

$$\mu_{I}=\frac{m}{K^{2}},$$ (29)

yielding $\mu_{I}=0.44\;{\text{electrons}}^{2}/s$. The y-intercept b of the plot determines the read noise because it corresponds to the noise level at zero exposure time, where dark current has not built up. The standard deviation of the read noise is

$$\sigma_{r}=\frac{\sqrt{b}}{K},$$ (30)

giving a read noise of $\sigma_{r}=14.1$ electrons.

## Code, Data, and Materials Availability

Code will be made available upon reasonable request.
